# Functional genomics of *hsp-90* in parasitic and free-living nematodes

**DOI:** 10.1016/j.ijpara.2009.02.024

**Published:** 2009-08

**Authors:** Victoria Gillan, Kirsty Maitland, Gillian McCormack, Nik A.I.I. Nik Him, Eileen Devaney

**Affiliations:** Parasitology Group, Division of Infection and Immunity, Institute of Comparative Medicine, Faculty of Veterinary Medicine, University of Glasgow, Bearsden Road, Glasgow G61 1QH, UK

**Keywords:** *Caenorhabditis elegans*, *Haemonchus contortus*, *Brugia pahangi*, Mutant rescue, RNAi

## Abstract

Heat shock protein 90 (Hsp-90) is a highly conserved essential protein in eukaryotes. Here we describe the molecular characterisation of *hsp-90* from three nematodes, the free-living *Caenorhabditis elegans* (Ce) and the parasitic worms *Brugia pahangi* (Bp) and *Haemonchus contortus* (Hc). These molecules were functionally characterised by rescue of a *Ce-daf-21* (*hsp-90*) null mutant. Our results show a gradient of rescue: the *C. elegans* endogenous gene provided full rescue of the *daf-21* mutant, while *Hc-hsp-90* provided partial rescue. In contrast, no rescue could be obtained using a variety of *Bp-hsp-90* constructs, despite the fact that *Bp-hsp-90* was transcribed and translated in the mutant worms. *daf-21* RNA interference (RNAi) experiments were carried out to determine whether knock-down of the endogenous *daf-21* mRNA in N2 worms could be complemented by expression of either parasite gene. However neither parasite gene could rescue the *daf-21* (RNAi) phenotypes. These results indicate that factors other than the level of sequence identity are important for determining whether parasite genes can functionally complement in *C. elegans*.

## 1 Introduction

Genome sequencing projects are providing a wealth of information on parasitic nematodes. The draft sequence of the *Brugia malayi* genome has been published (Ghedin et al., 2007), the *Haemonchus contortus* genome is nearing completion (900 Mb sequenced) and sequencing projects are planned for several other species of veterinary nematode (http://www.sanger.ac.uk/Projects/Helminths/). The comparative analysis of multiple parasitic nematode genomes has the capacity to further our understanding of the many adaptations associated with parasite life cycles (Wasmuth et al., 2008), and has the potential to facilitate the development of novel therapeutics. However, having the genome sequence available is only one element of a functional genomics approach; having the tools to define gene function is equally important. In the free-living nematode *Caenorhbditis elegans* a variety of methods have been developed to study gene function, including mutagenesis, RNA interference (RNAi) and transgenesis, as well as whole genome resources such as the interactome and promoterome projects (Dupuy et al., 2007; Hunt-Newbury et al., 2007). In contrast, the development of such methods for parasitic nematodes is in its infancy. Several laboratories have reported the efficacy of RNAi in parasitic nematodes (Hussein et al., 2002; Urwin et al., 2002; Aboobaker and Blaxter, 2003; Lustigman et al., 2004; Bakhetia et al., 2005; Islam et al., 2005; Issa et al., 2005; Geldhof et al., 2006), but the success of RNAi varies enormously with parasite species, life cycle stage used and genes targeted. Significant progress has been made in developing transgenesis for *Parastrongyloides trichosuri* (Grant et al., 2006) and *Strongyloides stercoralis* (Li et al., 2006; Junio et al., 2008) but not yet for other species.

An alternative approach for defining gene function of parasitic species is to use *C. elegans* as a heterologous expression system. One such method involves introducing the gene of interest into an appropriate *C. elegans* mutant and determining whether the parasite gene can restore the wild type (WT) phenotype. The majority of successful interspecies rescue experiments reported to date have come from work using *H. contortous*, a trichostrongyloid nematode parasite of sheep. *H. contortus* and *C. elegans* belong to the same Clade (V) and although these species are estimated to have diverged some 400 million years ago (Vanfleteren et al., 1994), *H. contortus* is one of the parasitic species most closely related to *C. elegans*. The *H. contortous* isotype 1 Β tubulin gene (*tub-1*) was the first parasite gene to be expressed in *C. elegans*; here the WT copy of the *H. contortus* gene was shown to confer sensitivity to benzimidazole (BZ) compounds in *ben-1* mutants which are BZ-resistant (Kwa et al., 1995). Other studies have demonstrated that an *H. contortous* cathepsin protease (*Hc*-*cpl-1)* can rescue the embryonic lethal phenotype of a *C. elegans cpl-1* mutant and also complement the *cpl-1(RNAi)* phenotype in WT worms (Britton and Murray, 2002). In addition, ectopic expression of a GATA transcription factor, *elt-2*, from *H. contortus* in *C. elegans* was shown to mimic the phenotype of endogenous over–expression (Couthier et al., 2004). Outside the Clade V nematodes, a forkhead transcription factor FKTF-1b, from *S. sterocoralis*, a Clade IV species, was recently shown to function in *C. elegans* and complement a *daf-16* mutant (Massey et al., 2006).

In this paper we present our attempts to rescue a *C. elegans* *daf-21*(*Ce-daf-21*), heat shock protein 90 (*hsp-90*) mutant with *hsp-90* from the filarial nematode *Brugia pahangi.* *B. pahangi* is a Clade III species and is much more distantly related to *C. elegans* than the trichostrongyles, such as *H. contortus.* However, the sequence of *hsp-90* is highly conserved between different nematodes and gene ontology predictions suggest conservation of Hsp-90 function. In mammalian cells and in yeast Hsp-90 is well characterised (Wandinger et al., 2008), functioning as a molecular chaperone to a sub-set of proteins many of which act in signal transduction pathways (Picard, 2002). Because of the nature of its client proteins Hsp-90 is essential in most organisms, including nematodes. For example, *Ce-daf-21* null mutants arrest at the L2/L3 stage, while worms carrying a single point mutation in *Ce-daf-21,* a weak gain of function mutation, are dauer-constitutive (Birnby et al., 2000). RNAi studies have also described a role for Hsp-90 in adult worms, as injection of double-stranded RNA resulted in cessation of egg production and an embryonic lethal phenotype (Piano et al., 2000; Inoue et al., 2006). Similarly, inhibition of Hsp-90 function in *B. pahangi* using the specific inhibitor Geldanamycin is lethal to all life cycle stages (Devaney et al., 2005). Given its genetic tractiblity and ease of manipulation, *C. elegans* has proved a useful model for studying the functions of parasite genes. In this paper we report our experiments aimed at rescuing a *Ce-daf-21* mutant with *hsp-90* from the parasitic species *B. pahangi* and comparative studies with *H. contortus*.

## 2 Materials and methods

### 2.1 Worm strains and molecular characterisation of C. elegans daf-21 mutants

*Caenorhabditis elegans* strains were cultured by standard methods (Sulston and Hodgkin, 1988) on normal growth medium (NGM) plates at 20 ^o^C, unless otherwise stated. Strains used were the N2 Bristol WT strain and LL1009 *daf-21*(*nr2081*)/*nT1[unc-?*(*n754*) *let-?*](IV;V) (originally published as LL1008 in Birnby et al. (2000)) both obtained from Caenorhabditis Genetics Centre (CGC). FX03133 *daf-21*(*tm3133*)(V) was obtained from National Bioresource Project (NBP), Japan. *daf-21(nr2081)* contains an 860-bp deletion plus a 3-bp insertion which is predicted to remove amino acids 32–287 and to add 94 novel amino acids from another reading frame, yielding the null allele. These worms carry a chromosomal balancer that confers an uncoordinated phenotype; heterozygotes are uncoordinated (Unc) and segregate Unc (heterozygotes), arrested aneuploids (balanced homozygotes) and non-Unc arrested as L2–L3 (deletion homozygotes). This allows the line to be maintained as a heterozygote with easy identification of deletion homozygotes and confirmation by diagnostic PCR using the primers LL1009 F1, LL1009 F2, LL1009 R1 (see Table 1). The WT allele amplifies two products of 1.2 kb and 330 bp while the deletion allele produces a PCR product of 430 bp.

The *daf-21*(*tm3133*) deletion allele results from a 479-bp deletion and a 4-bp insertion in *daf-21* with the deletion of amino acids 286–704 and the addition of 259 novel amino acids, generating the null allele. *daf-21*(*tm3133*) is not a balanced line and has to be selectively maintained as a heterozygote by single worm PCR using diagnostic primers Tm3133 F1, Tm3133 F2, Tm3133 R1 (see Table 1). Phenotypically WT heterozygotes segregate as follows: homozygous WT, heterozygotes and deletion homozygotes which arrest at L2/L3 stage. The WT allele amplifies two products of 1 kb and 250 bp, while the deletion allele produces a PCR product of 525 bp. Both mutant strains were back-crossed six times with WT *C. elegans*.

### 2.2 Sequence of nematode hsp-90s

The complete sequence for *Ce-daf-21* (C47E8.5) was available at Wormbase (http://www.wormbase.org/) while *B. pahangi hsp-90 (Bp-hsp-90,* AJ005784*)* was sequenced previously (Thompson et al., 2001). The full-length sequence for *H. contortus hsp-90* (*Hc-hsp-90,* FJ717747) was not available, so a tBLASTn search was carried out using the *Ce-*DAF-21 sequence against *H. contortus* combined reads at http://ubiquitinmine.nu/blast/blast.html/. A partial nucleotide sequence was obtained and the remainder of the gene was obtained by amplifying a fragment from *H. contortus* genomic DNA using the primers haem-541i08.q1 kb and haem-541i08.p1 kb, designed using the tools at http://ubiquitin.mine.nu/cgi-bin/getseq.cgi (see Table 1). The 2 kb fragment amplified by PCR was cloned into a pCR2.1 TOPO vector (Invitrogen), sequenced and used in a BLAST search against *H. contortus* combined reads, to identify the initiation methionine*.* Primers were designed to the 5′ and 3′ end of the gene (Hc-hsp-90 ORF F and Hc-hsp-90 ORF R, Table 1) and the full-length genomic sequence was obtained by PCR, cloned into a pCR2.1 TOPO vector and sequenced.

### 2.3 Transcriptional reporter gene constructs

The *LacZ* fusion vector pPD96.04 (Fire et al., 1990) was used to create a transcriptional reporter construct containing 4.1 kb of *Ce-daf-21* upstream sequence or a shorter 2.3 kb region. The promoter regions were amplified by PCR from WT *C. elegans* genomic DNA using the primers C47E8.5 4 kb prom BF or C47E8.5 2.3 kb prom F and C47E8.5 prom R (detailed in Table 1). The fragments were cloned into the intermediate vector pGEM-TEasy (Promega) and sub-cloned into pPD96.04 using the vector restriction sites SphI and PstI*.* Standard transgenic arrays have been shown to function well in somatic cells but not in the germline (Kelly et al., 1997) where *Ce*-*daf-21* is highly expressed. Germline silencing can be overcome by injecting a linearised transgene in a complex free array containing excess *C. elegans* genomic DNA. Therefore, purified plasmid DNA was treated with *Fsp*I, digesting the vector backbone at position 6534. The linearised construct was injected into the syncytial gonad of WT *C. elegans* worms as a complex array at a final concentration of 2 μg/ml. The injection mix also contained 60 μg/ml of *Pvu*II digested WT *C. elegans* genomic DNA and linearised pRF-4, which contains a dominant mutant allele of the *rol-6* gene (Kramer et al., 1990) as a marker of transgenesis. Lines displaying the roller phenotype were analysed for promoter activity by detection of β-galactosidase activity as described below.

### 2.4 Translational fusion with Bp-hsp-90

To confirm that *Bp-hsp-90* could be expressed in *C. elegans*, the *LacZ* fusion vector pPD95.03 (Fire et al., 1990) was used to create a translational reporter construct. The *Ce-daf-21* 4.1 kb promoter sequence was excised from the transcriptional promoter construct described above using SphI and PstI and ligated directly into pPD95.03*.* *Bp-hsp-90* was amplified from *B. pahangi* genomic DNA using AJ005784 ORF F and AJ005784 (95.03) ORF R primers (detailed in Table 1). The fragment was ligated into the intermediate vector pGEM-T Easy then sub-cloned into pPD95.03 via a restriction digest with *PstI*. Purified plasmid DNA was digested with *Fsp*I (digesting the vector backbone at position 5652) and injected at a final concentration of 2 μg/ml as a complex array together with pRF4 (as above). Lines displaying the roller phenotype were examined for *LacZ* expression as described below.

### 2.5 LacZ staining

Worms were washed off plates in M9 buffer (Brenner, 1974), washed on ice several times and then fixed with glutaraldehyde at a final concentration of 1.25% in distilled water for 20 min at room temperature. The fixed worms were washed with M9 buffer and pipetted onto a glass slide, air dried and dehydrated with acetone for 5 min at −20 ^o^C. The stain mixture contained 500 μl 0.4 M Na_3_PO_4_, 1 μl 1 M MgCl_2_, 100 μl Redox buffer, 4 μl 1% SDS, 400 μl H_2_O and 12 μl 2% X-Gal. Stain mix (50 μl) was added to the worms and the cover-slip sealed with nail varnish and placed in a humid chamber. Staining was checked every hour. Images were captured with a Zeiss Axioskop 2 Plus microscope using Openlab 3.1.4 software (Perkin–Elmer).

### 2.6 Rescue constructs

A number of different constructs were prepared for *Ce-daf-21, Bp-hsp-90* and *Hc-hsp-90* as detailed below*.* The constructs were individually assembled in a pBluescript SK^−^ vector. The expression cassette included 4.1 kb of upstream *Ce-daf-21* promoter sequence and 0.5 kb of *Ce-daf-21* 3′ untranslated region (UTR). C47E8.5 4 kb prom F and C47E8.5 prom R were used to amplify the promoter region while C47E8.5 3′UTR F and C47E8.5 3′UTR R were used to amplify the 3′UTR from *C. elegans* genomic DNA (see Table 1).

To create the *C. elegans* rescue construct, primers C47E8.5 ORF F and C47E8.5 ORF R (Table 1) were used to amplify *Ce-daf-21* from genomic DNA and cloned into the expression cassette. The *B. pahangi* and *H. contortus* rescue constructs were created as above using the same expression cassette to mimic the expression pattern of the endogenous protein as closely as possible. *Bp-hsp-90* was amplified by PCR from genomic DNA using primers AJ005784 ORF F and AJ005784 ORF R primers (Table 1). *Hc-hsp-90* was amplified from genomic DNA using the Hc-hsp-90 ORF F and Hc-hsp-90 ORF R primers (Table 1). The individual *hsp-90* PCR products were cloned into a pCR2.1 TOPO vector and cloned into a pBluescript SK^−^ expression vector. All rescue constructs were cut with *Fsp*I, digesting the pBluescript SK^−^ DNA backbone at sites 480 and 2269. Constructs were injected into the syncytial gonad as complex arrays at a final concentration of 2 μg/ml along with 60 μg/ml of *Pvu*II digested WT *C. elegans* genomic DNA and 2 μg/ml of *Fsp*I digested *dpy-7::GFP* (Roberts et al., 2003). Transformants were selected based on GFP expression and rescued homozygous mutants were identified on plates that contained only GFP-positive worms exhibiting the WT phenotype. Diagnostic PCR was carried out on *daf-21(nr2081)* expressing the *Bp-hsp-90* and *Hc-hsp-90* transgenes but *daf-21(nr2081)* expressing the *C. elegans* construct could not be genotyped by PCR as the transgene would have been amplified by the diagnostic primers.

In additional experiments *Bp-hsp-90* cDNA plus or minus a synthetic intron (SI) was injected into *daf-21(nr2081)* as a standard transgenic array. The SI (5′-GTAAGTTTAAACTATTCGTTACTAACTAACTTTAAACATTTAAATTTTCAG–3′) was inserted into the *Bp-hsp-90* cDNA construct by ligating the double-stranded oligo into an *Afe*I blunt-ended restriction site at position 1881. The *Bp-hsp-90* cDNA rescue constructs were injected at a range of concentrations, 25 μg/ml, 10 μg/ml and 2 μg/ml together with 2 μg/ml of *dpy-7::GFP* to identify transformants (Roberts et al., 2003). Injection mixes were made up to a final concentration of 150 μg/ml with pBluescript SK^−^.

### 2.7 Ce-daf-21 RNA interference

In addition to rescue of genetic mutants, attempts were also made to rescue the *daf-21(RNAi)* phenotypes by expression of a parasite *hsp-90*. To this end, transgenic lines of *C. elegans* were created carrying the *Bp-hsp-90* or *Hc-hsp-90* complex arrays in a WT background. For the *daf-21(RNAi)* construct, a 294 bp sequence at the 3′ region of *Ce-daf-21* was identified which had least homology to *Bp-hsp-90* and *Hc-hsp-90* (67% and 75% identical, respectively). This sequence was amplified from WT *C. elegans* cDNA using the primers C47E8.5 RNAi F and C47E8.5 RNAi R (Table 1) and cloned into a pCR2.1 TOPO vector before sub-cloning into the T7 double vector, L4440 via *Spe*I and *Xho*I restriction sites. The reaction was then transformed into competent HT115 (DE3) cells, an RNase III-deficient *Escherichia coli* strain with isopropyl-beta-d-thiogalactopyranoside-inducible T7 polymerase activity. Control plates were seeded with HT115 cells transformed with empty vector and *daf-21(RNAi)* was carried out using the standard bacterial feeding protocol (Timmons et al., 2001). Typically, four L4 were plated out in triplicate on *daf-21(RNAi)* or control plates, allowed to lay eggs and phenotypes scored in the F1 generation. Experiments were carried out at least three times at 16 ^o^C, 20 ^o^C and 25 ^o^C to assess penetrance of phenotypes at different temperatures.

### 2.8 RT-PCR of hsp-90

Total RNA was extracted from worms using Trizol (Invitrogen) according to the manufacturer’s protocol. RNA was stored in diethyl pyrocarbonate-treated H_2_O at −70 °C until use. Reverse transcription (RT) was carried out as described previously (Gillan and Devaney, 2005). Gene-specific primers were used in PCR reactions to identify the presence of *Hc-hsp-90* and *Bp-hsp-90* mRNA in transgenic lines. A 1.3 kb region of *Ce-daf-21* was amplified by PCR using primers C47E8.5 RT-PCR F and C47E8.5 RT-PCR R, a 600 bp region of *Bp-hsp-90* was amplified using primers AJ005784 RT-PCR F and AJ005784 RT-PCR R and a 1.34 kb fragment of *Hc-hsp-90* was amplified using primers Hc-hsp-90 RT-PCR F and Hc-hsp-90 RT-PCR R (see Table 1).

### 2.9 Immunolocalisation of DAF-21

DAF-21 expression was investigated in freeze cracked WT *C. elegans*. Gravid worms were dissected in M9 buffer containing 10 mM levamisole to release gut, gonad and embryos (Bobinnec et al., 2000). Antibody and nuclear staining was carried out essentially as described by Kinnaird et al. (2004) using the AC88 monoclonal antibody which recognises Hsp90 kindly provided by D.O. Toft (Mayo Clinic College of Medicine, Rochester, USA). AC88 was used at a dilution of 1:400 and was detected using anti-mouse Alexa 488 at 1:200. Specimens were mounted in 90% glycerol/DAPI.

### 2.10 Microscopy

Animals were mounted on 2% agarose containing 0.065% sodium azide on glass slides. All images, unless otherwise stated, were captured with a Zeiss Axioskop 2 Plus microscope using Openlab 3.1.4 software (Perkin Elmer).

### 2.11 Geldanamycin pull-downs

Geldanamycin (GA) is a specific inhibitor of Hsp90 that can be complexed to a solid support and used in pull-down assays to isolate GA-binding Hsp90. GA pull-downs were carried out exactly as described previously (Devaney et al., 2005). WT *C. elegans* expressing the *Bp-hsp-90* translational fusion, WT *C. elegans* and adult *B. pahangi* were lysed and 300 μg of each extract incubated with GA beads or control beads lacking GA. Bound products were eluted by boiling in SDS–PAGE sample cocktail and then analysed on 10% gels followed by immuno-blotting using a 1:5000 dilution of an antibody raised to *Bp-*Hsp-90. Bound antibody was detected using 1:10,000 dilution of anti-rabbit IgG (Sigma) followed by chemiluminescence.

### 2.12 Sequencing

The integrity of all the constructs was confirmed using automated DNA sequencing using vector and gene-specific primers (MWG Biotech).

## 3 Results

### 3.1 DAF-21 is expressed ubiquitously in C. elegans

**Fig. 1:**
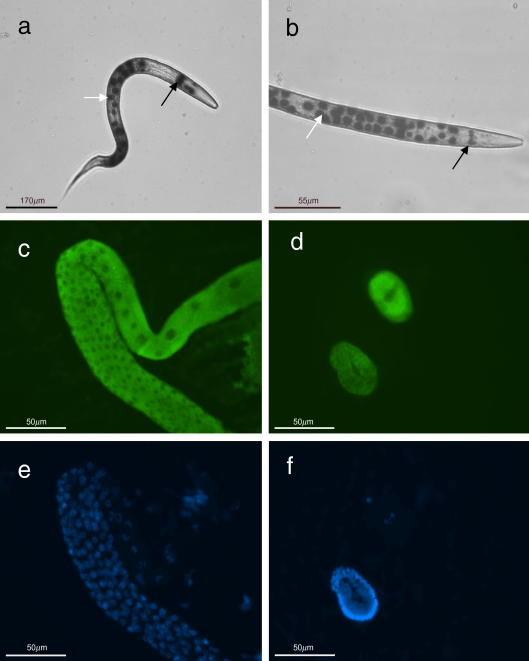
The *Caenorhabditis elegans daf-21* (*Ce-daf-21*) 4.1 kb promoter is active throughout the life cycle of *C. elegans*, while antibody staining shows Ce-DAF-21 is expressed in the germline. Wild type (WT) *C. elegans* worms were transfected with the 4.1 kb *Ce-daf-21* transcriptional fusion construct in pPD96.04 and stained with X-Gal for 2 h to identify β-galactosidase activity. (a) An L2 at 10× magnification. Scale bar is equal to 170 μm. (b) The head of an L4 at 40× magnification. Scale bar is equal to 55 μm. In (a) and (b), white arrows denote gut cells, black arrows denote nerve ring. (c–f) Gravid WT *C. elegans* hermaphrodites were freeze cracked and stained with 1:400 dilution of AC88 monoclonal antibody followed by Alexa 488 at 1:200 dilution. Staining is observed in the germline (c) and developing embryos (d) of WT *C. elegans* worms. The same samples stained with DAPI are shown in (e) and (f). (c)–(f) were captured at 40× magnification. Worms were viewed on a Zeiss Axioskop 2 Plus and images captured using Openlab 3.1.4 software. In (c)–(f), scale bars are equal to 50 μm.

In order to investigate the temporal and spatial expression pattern of *daf-21* *in C. elegans*, transcriptional reporter gene constructs containing either 2.3 kb or 4.1 kb of *daf-21* upstream sequence were generated. Analysis of β-galactosidase staining in these worms demonstrated that both promoters drive similar patterns of gene expression in all life cycle stages. However, in transgenic worms carrying the longer promoter, GFP fluorescence was also observed which was not apparent using the shorter promoter. For this reason, the longer promoter was used in all subsequent constructs. As shown in Fig. 1a and b, staining was evident in most somatic cells of the worm and was particularly prominent in the large gut cells. Staining was also observed in the nerve ring (arrow, Fig. 1a and b) and in neurones (data not shown). Previous studies have indicated that DAF-21 may be very highly expressed in the germline of *C. elegans* (Inoue et al., 2006). AC88, a well-characterised Hsp-90 monoclonal antibody that cross-reacts with *C. elegans* DAF-21, was used to stain freeze-cracked worms. Very high levels of expression were observed in the gonad and early embryos, suggesting that *daf-21* is maternally derived in *C. elegans* (Fig. 1c and d). Expression was also observed in additional tissues including muscle and possibly neurones in the head (data not shown).

### 3.2 Injection of Bp-hsp-90 in a complex array fails to rescue the daf-21 mutant phenotype

**Fig. 2:**
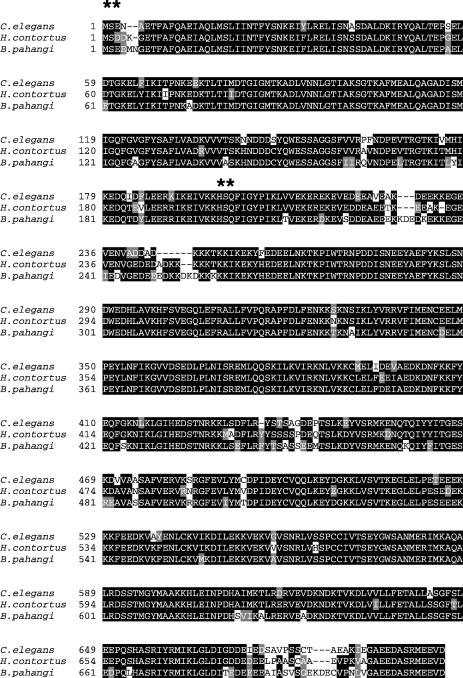
Heat shock protein 90 (Hsp-90) is highly conserved in *Caenorhabditis elegans* (*Ce*)*, Brugia pahangi (Bp)* and *Haemonchus contortus (Hc)*. The figure shows a Boxshade plot of the predicted amino acid sequences of all three nematode Hsp-90s. Identical amino acids are dark-shaded, similar are in grey. *Ce-*DAF-21 and *Bp-*Hsp-90 are 84% identical (91% similar), *Ce-*DAF-21 and *Hc-*Hsp-90 are 88% identical (93% similar) and *Bp-*Hsp-90 and *Hc-*Hsp-90 are 87% identical (93% similar) ∗∗ denotes the ATP-binding domain of Hsp-90.

Comparison of the nucleotide and predicted amino acid sequences of Hsp-90 from *C. elegans*, *B. pahangi* and *H. contortus* showed a high degree of conservation between all three sequences (see Fig. 2 for an amino acid alignment). To investigate the degree of functional conservation between *Bp-hsp-90* and *Ce-daf-21*, we attempted to rescue *daf-21(nr2081)* worms using constructs in which the transgene was embedded in a complex array. To first confirm that *daf-21(nr2081)* worms could be rescued by the endogenous *C. elegans* gene, the *daf-21* genomic sequence was cloned into a rescue plasmid (as described in Section 2) and injected as a complex array. In these experiments *dpy-7::gfp* was co-injected as a marker for transgenesis. As expected, the *C. elegans* construct rescued the L2/L3 arrest phenotype of the *daf-21(nr2081)* mutant in three lines studied. Twenty-five homozygous *daf-21(nr2081)* mutant worms containing the *Ce-daf-21* construct were plated out from each line and compared with WT *C. elegans* controls. All rescued worms were fertile and produced brood sizes comparable with WT *C. elegans* controls (data not shown).

**Fig. 3:**
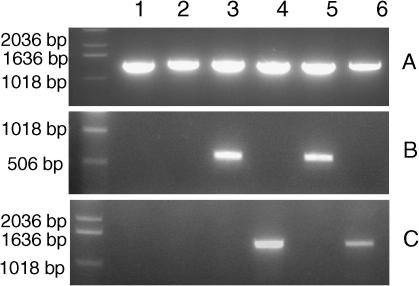
Transgenic *Caenorhabditis elegans* express parasite *hsp-90* mRNAs. Reverse transcriptase (RT)-PCR was carried out on wild type (WT) *C. elegans* worms (lane 1), *daf-21(nr2081)* worms (lane 2), WT *C. elegans* transfected with *Bp-hsp-90* complex array (lane 3), WT *C. elegans* transfected with *Hc-hsp-90* complex array (lane 4), *daf-21(nr2081)* transfected with *Bp-hsp-90* complex array (lane 5) and *daf-21(nr2081)* transfected with *Hc-hsp-90* complex array (lane 6) using gene-specific primers for *Ce-daf-21* (A), *Bp-hsp-90* (B) and *Hc-hsp-90* (C)*.* PCR products were resolved on a 1% agarose gel.

The *Ce-daf-21* genomic sequence was replaced with the *Bp-hsp-90* genomic DNA and the resulting plasmid injected into *daf-21(nr2081)* hermaphrodites as a complex array at a concentration of 2 μg/ml. One hundred worms from each of three lines were cloned and examined by microscopy. Rescue of mutant animals (defined by F1 animals exhibiting GFP expression and appearing WT, having lost the balancer and the Unc phenotype) was never observed. To confirm this result, a further 100 GFP-positive *daf-21(nr2081)* adult worms from each line were cloned, allowed to lay eggs and the single adult worm genotyped by PCR using the diagnostic primers. Homozygous mutant worms expressing the transgene never displayed a rescued phenotype. All F1 worms which reached adulthood were Unc and PCR reactions on single worms gave products of 1.2 kb and 330 bp, a pattern consistent with a heterozygotic genotype. Confirmation that *Bp-hsp-90* mRNA was expressed from the complex array in *daf-21* mutant worms was obtained by RT-PCR (Fig. 3b, lane 5).

Examination of the genomic structure of the three nematode *hsp-90*s reveals significant differences: *Bp-hsp-90* contains 11 introns, while *Ce-daf-21* and *Hc-hsp-90* contain three and eight introns, respectively (data not shown). In case the *B. pahangi* genomic DNA transgene was not properly spliced in *C. elegans*, we generated additional rescue constructs containing either the *Bp-hsp-90* cDNA, with or without a synthetic intron. Constructs were prepared using the same promoter and 3′ UTR as described previously and injected as standard transgenes using DNA at a concentration of 25 μg/ml. However, in all cases it was difficult to obtain transgenic lines so DNA was injected at 10 μg/ml or 2 μg/ml. Although multiple lines were obtained with the 2 μg/ml injection mix, on no occasion was rescue successful as defined by phenotypic observation or the diagnostic PCR described above.

As an alternative to rescuing the mutant phenotype of *daf-21(nr2081)* worms, the *Bp-hsp-90* complex array was injected into WT *C. elegans* worms and a number of lines established for crossing into an alternative deletion mutant strain, *daf-21*(*tm3133*). *daf-21*(*tm3133*) heterozygous hermaphrodites were crossed with transgenic *C. elegans* males containing the *Bp-hsp-90* complex array. One hundred *daf-21*(*tm3133*) GFP adult worms from each line were cloned, allowed to lay eggs and phenotypes observed. Complementation of the homozygous deletion mutant would result in F1 animals exhibiting GFP expression. A further 100 *daf-21*(*tm3133*) GFP-positive adult worms were cloned from each line, allowed to lay eggs and single worm PCR carried out to genotype the P0 animal. PCR products of 1 kb and 250 bp were amplified from all P0 animals, a pattern consistent with heterozygote controls (data not shown).

### 3.3 Bp-Hsp-90 is expressed in C. elegans

**Fig. 4:**
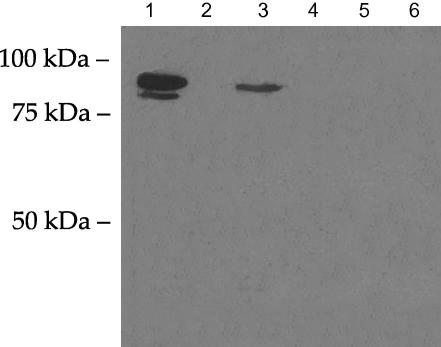
*Brugia pahangi* heat shock protein 90 (*Bp-hsp-90*) is translated in *Caenorhabditis elegans* and binds Geldanamycin (GA). Adult *B. pahangi* (lanes 1 and 4), wild type (WT) *C. elegans* worms (lanes 2 and 5) or WT *C. elegans* expressing the *Bp-hsp-90* translational fusion (lanes 3 and 6) were lysed and used in a GA pull-down. Each extract (300 μg) was incubated with GA beads (lanes 1–3) or control beads (lanes 4–6) and pull-downs analysed by SDS–PAGE and blotting with an anti-Hsp-90 antibody at 1:5000 dilution followed by goat anti-rabbit secondary antibody at 1:10,000 dilution.

As we could not obtain a rescue line using a variety of *Bp-hsp-90* constructs, it was important to determine whether the *B. pahangi* transgene was efficiently translated in *C. elegans*. For this purpose, we first prepared a translational construct containing the *B. pahangi* genomic sequence under the control of the *C. elegans* 4.1 kb promoter and cloned this into pPD95.03. This was injected into WT *C. elegans* worms as a complex array together with linearised *pRF4* and the pattern of expression examined by staining for β-galactosidase. The staining was very similar to that observed with the transcriptional reporters as shown in Fig. 1a and b demonstrating that the *B. pahangi* gene is transcribed in most tissues in *C. elegans*. As an additional test of translation, we also carried out a pull-down using GA conjugated to a solid support. *Ce-*DAF-21 does not bind to GA, in contrast to *Bp-*Hsp-90. Fig. 4 shows the results of a representative pull-down, with WT *C. elegans* worms expressing the translational fusion giving a positive signal with GA beads (lane 3), while WT *C. elegans* do not (lane 2). Similar results were obtained with a second line of transfected worms (data not shown).

### 3.4 Injection of an Hc-hsp-90 construct provides partial rescue of a Ce-daf-21 mutant

**Fig. 5:**
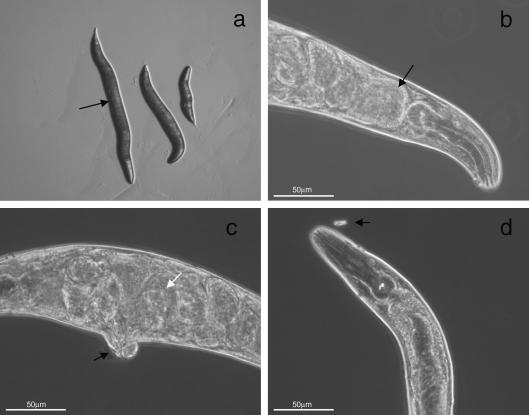
Expression of *Haemonchus contortus* heat shock protein 90 (*Hc-hsp-90*) provides partial rescue of the mutant *daf-21(nr2081)*. *daf-21(nr2081)* mutants were transfected with the *Hc-hsp-90* complex array and single worm PCR was carried out to identify homozygous mutant worms surviving past the L2/L3 arrest. (a) An adult wild type (WT) *Caenorhabditis elegans* (arrow) next to two partially rescued worms showing the variation in size and extremely dumpy phenotype of the partial rescue. Worms were aged matched. Plate images were viewed on a Zeiss Stemi SV6. (b) The highly distended gut, packed with bacteria (arrow) and (c), the grossly abnormal embryo arrangement (white arrow) and protruding vulva (black arrow). (d) A partially rescued worm displaying an improperly shed pharyngeal cuticle (arrow). Worms were viewed on a Zeiss Axioskop 2 Plus microscope at 40× magnification and images captured using Openlab 3.1.4. Scale bars on each image represent 50 μm.

As there are no published reports, to our knowledge, in which filarial genes have been used to rescue *C. elegans* mutants, it was difficult to ascertain whether the lack of rescue observed was a function of attempting to express a filarial gene or, perhaps more likely, a function of *Bp*-*hsp-90* itself. In an attempt to address this question we prepared a construct containing a genomic copy of *Hc-hsp-90* under the control of the same *Ce-daf-21* 4.1 kb promoter and 3′ UTR and injected this into *daf-21(nr2081)* worms as a complex array. The *Hc-hsp-90* construct was expressed in *daf-21(nr2081)* worms (Fig. 3c, lane 6) and conferred partial rescue of the mutant in all three lines. Deletion homozygotes (defined by diagnostic PCR) expressing the transgene developed past the L2/L3 arrest to adulthood. In the three lines examined in detail, 100% of the partially rescued worms were dumpy and were sterile. Fig. 5a shows the comparative morphology of a WT *C. elegans* with two partially rescued worms. In these dumpy worms the intestine and gonad were grossly distorted to fit within the body (Fig. 5b and c) and in 100% of these animals both the vulva and rectum were malformed and protruding. These worms contained embryos, but produced no viable progeny and larvae did not hatch within the adult worm, suggesting an embryonic lethal phenotype. In a proportion of these partially rescued animals, remnants of the pharyngeal cuticle remained attached, suggestive of a moult phenotype (Fig. 5d). In additional experiments, individual dumpy worms were plated out at different temperatures (16, 20 and 25 °C), but no progeny were observed irrespective of incubation temperature. These worms were confirmed to be homozygous deletion mutants by single worm PCR using diagnostic primers.

### 3.5 Ce-daf-21(RNAi) in Bp-hsp-90 and Hc-hsp-90 transgenic worms gives differing phenotypes

**Fig. 6:**
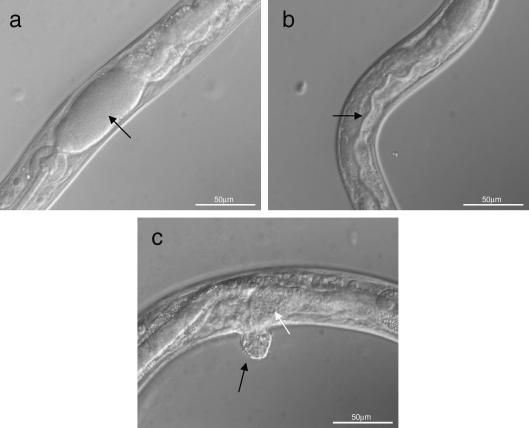
*Caenorhabditis elegansdaf-21* RNAi interference (*Ce-daf-21(RNAi*)) results in pronounced phenotypes and sterility in the F1 generation. Wild type (WT) *C. elegans* worms were grown at 25 ^o^C on normal growth medium (NGM) plates seeded with HT115 cells containing the L4440 feeding vector with the *Ce-daf-21* insert. (a–c) Nomarski imaging of adult WT *C. elegans* exposed to *daf-21(RNAi).* (a) The highly distended gut packed with bacteria (arrow), (b) the distorted alimentary canal and (c) the under-developed gonad (white arrow) and protruding vulva (black arrow). Worms were viewed on a Zeiss Axioskop 2 Plus microscope at 40× magnification and images captured using Openlab 3.1.4 software. Scale bars on each image represent 50 μm.

As an alternative to rescuing the mutant strain with constructs containing *Bp-hsp-90* or *Hc-hsp-90*, *daf-21(RNAi)* experiments were carried out in *C. elegans* to assess whether knock-down of the endogenous mRNA can be rescued by either parasite gene in WT *C. elegans* worms. As a prelude to these experiments, it was important to define the *daf-21(RNAi)* phenotypes in WT *C. elegans*. Initial studies were carried out at 16 ^o^C, 20 ^o^C or 25 ^o^C but as all phenotypes observed were most penetrant at higher temperatures, subsequent experiments were carried out at 25 ^o^C. P0 WT *C. elegans* grown on *daf-21(RNAi)* plates had normal brood sizes compared with worms grown on control plates, but the F1 generations were sterile, showed reduced mobility and had a distinctive protruding vulva (100% penetrant at 25 ^o^C). These worms also displayed a ‘distended gut’ phenotype in which the anterior part of the intestine was swollen and apparently blocked with bacteria (Fig. 6a and b) and showed little or no development of the gonad (Fig. 6c). *daf-21(RNAi)* was then carried out on WT *C. elegans* containing the *Bp-hsp-90* trangene, injected as a complex array. Expression of the *B. pahangi* transgene (confirmed by RT-PCR, Fig. 3b lane 3) failed to rescue any of the *daf-21(RNAi)* phenotypes. All F1 worms were sterile and indistinguishable from WT *C. elegans* *daf-21(RNAi)* worms.

**Fig. 7:**
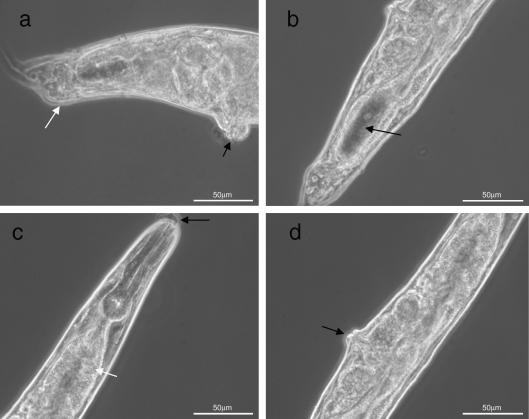
*Caenorhabditis elegansdaf-21* RNA interference (*Ce-daf-21(RNAi*)) in worms expressing *Haemonchus contortus* heat shock protein 90 (*Hc-hsp-90*) gives alternative phenotypes. Wild type (WT) *C. elegans* worms expressing the *Hc-hsp-90* complex array were grown on plates seeded with HT115 cells containing L4440 with the *Ce-daf-21* insert. *Hc-hsp-90* expressing worms display abnormal rectal formation (a, white arrow) and protruding vulva (a, black arrow), underdeveloped gonad (b, arrow), distended gut (c, white arrow) and inability to shed pharyngeal cuticle (c, black arrow) and protruding vulva (d, arrow). Worms were viewed on a Zeiss Axioskop 2 Plus microscope at 40× magnification and images captured using Openlab 3.1.4. Scale bars on each image represent 50 μm.

Similar *daf-21(RNAi)* experiments were then carried out on WT *C. elegans* worms expressing the *Hc-hsp-90* transgene injected as a complex array. Interestingly expression of the *H. contortus* transgene (confirmed by RT-PCR, Fig. 3c lane 4) resulted in different phenotypes from those observed in *daf-21(RNAi)*-treated WT *C. elegans* worms or in transformed *C. elegans* expressing the *Bp-hsp-90* construct. All *daf-21(RNAi)* worms expressing *Hc-hsp-90* were dumpy but very motile and appeared healthier than *daf-21(RNAi)*-treated WT *C. elegans*. However, 100% of F1 *daf-21(RNAi)* worms expressing *Hc-hsp-90* displayed abnormal rectal phenotypes (Fig. 7a), had an under-developed gonad (Fig. 7b), showed a distended gut (Fig. 7c), were sterile and formed a protruding vulva (Fig. 7d). These phenotypes were much less pronounced than in *daf-21(RNAi)*-treated WT *C. elegans* (compare Figs. 6c and 7d) or in worms expressing *Bp-hsp-90*. Additionally, under the compound microscope, shedding of the pharyngeal cuticle was seen to be incomplete in a proportion of *daf-21(RNAi)* worms expressing *Hc-hsp-90* (Fig. 7c). These phenotypes are notably similar to those observed in the partial rescue of the *daf-21* genetic mutant by *Hc-hsp-90*. All abnormal phenotypes observed were dependent on exposure to *daf-21(RNAi)*, as transgenic animals expressing *Bp-hsp-90* or *Hc-hsp-90* appeared WT on control plates (seeded with HT115 cells containing L4440 empty vector).

## 4 Discussion

In this paper we show that transformation of *daf-21(nr2081)* mutants with parasite *hsp90* provides a gradient of rescue; *Bp*-*hsp-90* cannot rescue the *C. elegans* mutant or complement *daf-21(RNAi)* worms, while transformation with *Hc*-*hsp-90* provides partial rescue. Given the degree of sequence similarity between the molecules from *C. elegans*, *B. pahangi* and *H. contortus*, each of which share 84% or greater identity at the amino acid level, the lack of rescue was surprising. For example, *hsp83* (the *hsp90* homologue) from *Trypanosoma cruzi* can complement yeast (Palmer et al., 1995), as can *Ce-daf-21* and human *hsp-90* β (Piper et al., 2003) despite significantly lower levels of identity (60.5% and 60.3%, respectively). However, the overall degree of sequence similarity may not be a good measure of function. When *H. contortus elt-2* was ectopically expressed in *C. elegans*, it was able to activate a programme of endodermal differentiation, demonstrating conservation of function. *Hc-elt-2* shares minimal sequence identity with *Ce-elt-2* (26.8% overall), although the DNA-binding domain is highly conserved (Couthier et al., 2004). Similarly, *fktf-1b*, the forkhead transcription factor from *S. stercoralis* that rescues a *C. elegans daf-16* mutant, shows a relatively low level of homology to *daf-16*, except over the DNA-binding domain which is 80% identical (Massey et al., 2006). In complex metazoans such as *C. elegans*, other factors may take precedence such as the timing, place and level of expression of an introduced transgene. However use of the *daf-21* promoter and 3′ UTR ought to mimic the spatial and temporal expression pattern of the endogenous protein. It was notable that constructs containing *Bp-hsp-90* had to be injected at significantly lower concentrations than normal to obtain transgenic lines, an observation that may reflect toxicity of the *B. pahangi* protein when over-expressed in *C. elegans*. The use of a strictly inducible promoter such as *hsp-16-2* might help overcome such limitations. Alternatively it may be possible to generate low-copy number chromosomal integrants using gamma or UV irradiation (Mello et al., 1991) or microparticle bombardment (Praitis, 2006).

The abundant expression of DAF-21 in the *C. elegans* germline posed a challenge for expression of the transgene from a standard array. Rescue of the mutant with the endogenous *Ce-daf-21* was only possible when introduced in a complex array, but neither parasite gene was fully functional in a similar construct. *Bp-hsp-90* mRNA was expressed in *C. elegans* as shown by RT-PCR analysis and predicted to be translated as evidenced by examination of the worms containing the *Bp-hsp-90* translational fusion and by the ability of *Bp*-Hsp-90 from these worms to bind to GA. In contrast to *B. pahangi*, partial rescue was obtained with *Hc-hsp-90*, a species more closely related to *C. elegans*. The *H. contortus* transgene presumably provided sufficient Hsp-90 to allow development to proceed beyond the L2/L3 arrest but not enough to provide a full rescue, although similar results were obtained with several lines. A further possibility is that the parasite transgenes may rescue somatic phenotypes, but not germline phenotypes, despite the use of complex arrays (Kelly et al., 1997). A method of transposon-based integration which allows expression of single copy transgenes from defined sites, including the germline, has recently been described (Frokjaer-Jensen et al., 2008) and may be useful in future studies.

In yeast, complementation by human *hsp-90* β and *Ce-daf-21* required the presence of the co-chaperone STI-1/Hop (hsp-organising protein in metazoans) (Piper et al., 2003), a finding that highlights the fact that Hsp-90 does not function in isolation but rather as part of a multi-protein complex. The complex can be functionally re-constituted in vitro and minimally requires five proteins: Hsp-40, Hsp-70, Hsp-90, Hop and p23, plus ATP for activity, although in vivo other proteins are also found in complexes (Pratt and Toft, 2003). The *C. elegans* genome contains likely homologues of Hop/STI-1 (R09E12.3) and p23 (ZC395.10), suggesting that the minimal mammalian complex is present. However, the different nematode Hsp-90s may require different co-chaperones for activity and while the basic complement of interacting proteins are present, perhaps an essential co-chaperone is missing from *C. elegans* so that the parasite transgenes fail to mediate some of the essential functions of DAF-21 in *C. elegans*. One notable Hsp-90 interacting protein absent from the *C. elegans* genome is the cyclophilin *cyp-40*. Only one large tetratricopeptide (TPR) – containing immunophilin, *fkb-6,* is found in the *C. elegans* genome*. fkb-6* interacts with the C-terminal domain of Hsp-90 but is largely restricted to neuronal tissues in the worm (Richardson et al., 2007). However there are additional proteins with TPR domains that interact with Hsp-90 in *C. elegans* (Worrall et al., 2008). Likewise in *B. malayi* there are a family of cyclophilin-like proteins (Ma et al., 2002), but no clear homologue of *cyp-40*. Alternatively, subtle differences in the nematode *hsp-90* sequences may be sufficient to alter the binding of key client proteins such that Hsp-90 function is compromised.

The results with the *daf-21* mutants were confirmed in *daf-21(RNAi)* experiments in WT *C. elegans*, in which the *B. pahangi* transgene failed to complement the *daf-21(RNAi)* knock-down. The *daf-21(RNAi)* phenotypes were similar to those observed previously (Piano et al., 2000; Inoue et al., 2006) with F1 worms being largely infertile. However additional phenotypes were observed including a swollen anterior gut, which appeared to be blocked with bacteria. A similar phenotype was previously reported in *hsf-1(RNAi)* worms (Garigan et al., 2002) where it was proposed to contribute to the reduced lifespan observed.

Although we have been unable to rescue the *C. elegans* mutant with a *B. pahangi* gene or to complement the *daf-21(RNAi)* knockdown, additional filarial genes could be tested to determine whether our results are a feature of *hsp-90* and its requirement for additional proteins for activity, or whether perhaps the evolutionary distance between *C. elegans* and *B. pahangi* limits this type of experiment. Previous studies have demonstrated that expression of an *Onchocerca volvulus* GST gene in *C. elegans* conferred increased resistance to oxidative stress and confirm that some filarial genes can be expressed and function in *C. elegans* (Kampkotter et al., 2003). In addition, as we (Thompson et al., 2001), and others (Krause et al., 2001; Gomez-Escobar et al., 2002; Winter et al., 2003), have previously shown that filarial promoters, including *hsp-90*, can drive reporter gene expression in *C. elegans*, although not always with the same pattern as the endogenous promoter, we believe that our results are more likely to reflect differences in the structure and function of Hsp-90 itself, or differences in client proteins/co-chaperone usage in parasitic and free-living nematodes. Despite a high degree of sequence homology, nematode Hsp-90s are functionally diverse as exemplified by the inability of *C. elegans* and *H. contortus* Hsp-90 to bind GA in contrast to *B. pahangi* Hsp-90 (Nik Him et al, unpublished data). Our on-going studies are focused on understanding the molecular basis of these differences in nematode Hsp-90s. Given the availability of the *B. malayi* genome, the development of methods for defining gene function will be essential to make best use of this resource.

## Figures and Tables

**Table 1:** Names and sequences of primers used in this study. Underlined sequences denote restriction sites. Initiation methionines are shown in bold.

Primer name	Primer sequence
LL1009 F1	5′-ATGTCCGAGAACGCCGAAACCTTCGCA-3′
LL1009 F2	5′-CGAAGAATATGCTGAGTTCTACAAG-3′
LL1009 R1	5′-GCTCCATGCACTTCTTGACAAGAT-3′
Tm3133 F1	5′-TCCTCGAGGAGCGCAAG-3′
Tm3133 F2	5′-TCGGAATCCACGAGGAC-3′
Tm3133 R1	5′-GAGTTGTTGGACGCAGTAC-3′
haem-541i08.q1 kb	5′-GTAGATAGTATCAAACCTCAGTA-3′
haem-541i08.p1 kb	5′-TACCGGTCGTGTATTACATCT-3′
C47E8.5 2.3 kb prom F	5′-CGCGGTACCCGAATGGTTCCCATCGTGAC-3′ corresponding to positions −2302 to −2276 relative to the ATG start codon (KpnI site underlined)
C47E8.5 4 kb prom F	5′-CGCGGTACCTGGGTATGTTAAAATCGGAATAGTTG-3′ corresponding to positions −4060 to −4028 relative to the ATG start codon (KpnI site underlined)
C47E8.5 prom R	5′-CGCCTGCAGGGTTCTGGAAAAATATC-3′ corresponding to positions −23 to −1 relative to the ATG start codon (PstI site underlined)
C47E8.5 ORF F	5′-CGCCTGCAG**ATG**TCCGAGAACGC-3′ (PstI site underlined)
C47E8.5 ORF R	5′-CGCGCGGCCGCTTAGTCGACCTCCT-3′ (NotI site underlined)
C47E8.5 3′UTR F	5′-GAGCGGCCGCGTTACGCGAAGATCTCC-3′ (NotI site underlined)
C47E8.5 3′UTR R	5′-GACCGCGGGATTAGACAGCAAGGTTTGG-3′ corresponding to positions + 487 to + 461 relative to TAA stop codon (SacII site underlined)
AJ005784 ORF F	5′-CGCCTGCAG**ATG**TCGGAAGAAATGAATGGTGAA-3′ (PstI site underlined)
AJ005784 ORF R	5′-GAGCGGCCGCTTAATCAACTTCTTCCATCCTCGA-3′ (NotI site underlined)
Hc-hsp-90 ORF F	5′-GCGCTGCAG**ATG**TCTGACGACAAAGGCGAAAC-3′ (PstI site underlined)
Hc-hsp-90 ORF R	5′-CGCGCGGCCGCCTAGTCGACCTCCTCCATTCGGGATG-3′ (NotI site underlined)
C47E8.5 RNAi F	5′-ATCAACCCAGACCACGCTATCATGAAG-3′
C47E8.5 RNAi R	5′-TTAGTCGACCTCCTCCATGCG-3′
C47E8.5 4 kb prom BF	5′-TGGGTATGTTAAAATCGGAATAGTTG-3′
C47E8.5 internal R	5′-TGCAATGTGGATGATGATCTACATC-3′
AJ005784 (95.03) ORF R	5′-GCGCTGCAGGCATCAACTTCTTCCATCCTCGAT-3′ (PstI site underlined)
AJ005784 internal R	5-TTCACAATCTCCTTGATGCGAC-3′
AJ005784 internal F	5′-TATAAATGTGGGAAGAGGA-3′
pPD95.03 R	5′-CACCCACCGGTACCTTAC-3′
C47E8.5 RT-PCR F	5′-TCGCTGACGACGCTGACAAGAAGA-3′
C47E8.5 RT-PCR R	5′-CGGTGCACGATGATGGAACAGCAGAAT-3′
AJ005784 RT-PCR F	5′-TGATCCGATAGACGAGTATTG-3′
AJ005784 RT-PCR R	5′-AACTAAGTTTGGCACACATTCGTCTTTCT-3′
Hc-hsp90 RT-PCR F	5′-GGACGAGGATGCCGATAAGAAG-3′
Hc-hsp90 RT-PCR R	5′-CTGCACAAGAAGCAGCAGGGAGT-3′
